# Announcements

**Published:** 2015-04-10

## National Public Health Week — April 6–12, 2015

Every year since 1995, the American Public Health Association has led the observation of National Public Health Week in the United States during the first full week of April. The goal of National Public Health Week is to acknowledge contributions made by public health and to raise awareness of issues important to improving the nation’s health. This year’s observance (April 6–12) focuses on making the United States the Healthiest Nation in One Generation by 2030. Additional information about this year’s observance is available at http://www.nphw.org.

In conjunction with this year’s observance, CDC is partnering with the American Public Health Association to promote daily themes for National Public Health Week, by sharing information on CDC topics that align with each day’s theme. Additional information available at http://www.cdc.gov/features/public-health-week/.

## National Infant Immunization Week — April 18–25, 2015

National Infant Immunization Week (NIIW) is April 18–25, 2015. This annual observance promotes the benefits of childhood immunizations and their role in improving the health of children aged ≤2 years. Since 1994, local and state health departments, immunization partners, health care professionals, community leaders, clinicians from across the United States, and CDC have come together to highlight the importance of vaccination in the lives of infants and children.

Although immunization coverage among children remains at high levels, recent outbreaks of measles in the United States highlight the importance of maintaining high immunization rates. NIIW provides an opportunity to celebrate immunization achievements, recognize partners and volunteers dedicated to childhood immunization, and revitalize community efforts to maintain high vaccination levels.

During NIIW, local and state health departments, national immunization partners, and health care professionals will host events and educational activities for parents and clinicians. To help with planning these activities, various promotional and educational materials are available from CDC on the NIIW website.* Also available are materials from CDC’s new *Born with Protection* campaign,† which promotes whooping cough vaccination during the third trimester of each pregnancy to help protect babies during their first few months of life when they are most vulnerable.

## Footnotes

* Additional information available at http://www.cdc.gov/vaccines/events/niiw/index.html.

† Additional information available at http://www.cdc.gov/pertussis/pregnant/index.html.

